# Functional analysis and binding affinity of tomato ethylene response factors provide insight on the molecular bases of plant differential responses to ethylene

**DOI:** 10.1186/1471-2229-12-190

**Published:** 2012-10-11

**Authors:** Julien Pirrello, BC Narasimha Prasad, Wangshu Zhang, Kunsong Chen, Isabelle Mila, Mohamed Zouine, Alain Latché, Jean Claude Pech, Masaru Ohme-Takagi, Farid Regad, Mondher Bouzayen

**Affiliations:** 1 INP-ENSA Toulouse, Université de Toulouse, GBF, Avenue de l′Agrobiopole BP 32607, Castanet-Tolosan, F-31326, France; 2 INRA, UMR990 Génomique et Biotechnologie des Fruits, Chemin de Borde Rouge, Castanet-Tolosan, F-31326, France; 3 Institute of Fruit Science, Zhejiang University, Hangzhou, 310029, China; 4 Research Institute of Genome-based Biofactory, National Institute of Advanced Industrial Science and Technology, Central 4, Tsukuba, 305-8562, Japan

**Keywords:** Ethylene, ERF, Transcriptional regulation, *cis*-regulatory elements, Tomato

## Abstract

**Background:**

The phytohormone ethylene is involved in a wide range of developmental processes and in mediating plant responses to biotic and abiotic stresses. Ethylene signalling acts via a linear transduction pathway leading to the activation of *Ethylene Response Factor* genes (*ERF*) which represent one of the largest gene families of plant transcription factors. How an apparently simple signalling pathway can account for the complex and widely diverse plant responses to ethylene remains yet an unanswered question. Building on the recent release of the complete tomato genome sequence, the present study aims at gaining better insight on distinctive features among ERF proteins.

**Results:**

A set of 28 cDNA clones encoding ERFs in the tomato (*Solanum lycopersicon*) were isolated and shown to fall into nine distinct subclasses characterised by specific conserved motifs most of which with unknown function. In addition of being able to regulate the transcriptional activity of GCC-box containing promoters, tomato ERFs are also shown to be active on promoters lacking this canonical ethylene-responsive-element. Moreover, the data reveal that ERF affinity to the GCC-box depends on the nucleotide environment surrounding this *cis*-acting element. Site-directed mutagenesis revealed that the nature of the flanking nucleotides can either enhance or reduce the binding affinity, thus conferring the binding specificity of various ERFs to target promoters.

Based on their expression pattern, *ERF* genes can be clustered in two main clades given their preferential expression in reproductive or vegetative tissues. The regulation of several tomato *ERF* genes by both ethylene and auxin, suggests their potential contribution to the convergence mechanism between the signalling pathways of the two hormones.

**Conclusions:**

The data reveal that regions flanking the core GCC-box sequence are part of the discrimination mechanism by which ERFs selectively bind to their target promoters. *ERF* tissue-specific expression combined to their responsiveness to both ethylene and auxin bring some insight on the complexity and fine regulation mechanisms involving these transcriptional mediators. All together the data support the hypothesis that ERFs are the main component enabling ethylene to regulate a wide range of physiological processes in a highly specific and coordinated manner.

## Background

The gaseous plant hormone ethylene is reported to play an active role in a wide range of developmental processes, including germination, flower and leaf senescence, fruit ripening, leaf abscission, root nodulation, programmed cell death, and responses to abiotic stresses and pathogen attacks [1-3]. Components of ethylene signalling have been extensively studied mainly in Arabidopsis model plant [4] revealing a linear transduction pathway that leads to the activation of transcriptional regulators belonging to the Ethylene Response Factor (ERF) type. These last components of the ethylene signalling pathway are responsible for modulating the transcription of ethylene-regulated genes. Whereas the apparent simplicity of the ethylene transduction pathway cannot account for the wide diversity of plant responses to ethylene, the molecular mechanisms that enable this hormone to drive different physiological and developmental processes in an appropriate way are yet to be elucidated. Being encoded by one of the largest family of plant transcription factors, ERF proteins are the most suited step of ethylene signalling where the diversity and specificity of ethylene responses may originate.

ERFs are *trans*-acting factors unique to plants shown to bind specifically to GCC box *cis*-acting elements found in the promoter regions of ethylene-responsive genes [5,6]. The ERF family is part of the AP2-containing domain superfamily which also contains the AP2 and RAV families of transcription factors [7]. The AP2 family is characterized by the presence of two copies of the AP2 domain initially described in the Arabidopsis homeotic gene *APETALA2*[8]. The RAV family contains two DNA binding domains, an AP2-like domain that binds the CAACA motif and a B3-like domain that binds the CACCTG motif [9]. The ERF type family is further divided into two major subfamilies, the ERF and the CBF/DREB (C-repeat binding factor/dehydration responsive element binding factor) subfamilies of transcription factors [10] both containing a single AP2 domain. DREB subfamily is characterized by the presence of a valine and glutamic acid respectively at position 14 and 19 in the AP2 domain, whereas alanine and aspartic acid are conserved in the corresponding positions for ERFs [10]. It has been demonstrated that the amino acid residues involved in DNA binding are not conserved between AP2 and ERFs [11,12]. ERF type proteins have been first isolated in the context of plant responses to biotic stress while DREB are associated with abiotic stress. ERFs have been shown to bind the GCC-box found in ethylene-responsive genes and DREBs to the DRE *cis*-regulatory element. In *Arabidopsis*, ERFs account for up to 53 % of the total ERF/DREB proteins [13]. In poplar and rice ERF proteins represent 54 % of the ERF/DREB family [13,14] whereas it represents 51 % in soybean [15]. Comparative structural analysis of Arabidopsis and rice ERFs, have been performed *in silico* using either the entire protein sequence for phylogenetic analysis [16,17], or the conserved AP2 domain [10,13] to infer relationship between ERF family members. In Arabidopsis the ERF subfamily contains 65 members and is divided into 5 subclasses based on the conservation of the AP2 domain [13]. The ERF domain, first identified as a conserved motif of 59 amino acids in four DNA-binding proteins from *Nicotiana tabacum*[5] is characterized by 3 β-sheets and 1 α-helix and allows binding of the ERFs to the GCC-box [5,11]. In addition to the requirement for a perfectly conserved GCC motif, it has been suggested that the binding affinity may also depend on the nucleotide environment of the GCC box as well as on the nature of some amino acid residues within the ERF binding domain [17]. Moreover, in line with the data reporting that *Pti4*, an ERF type transcription factor, is able to bind promoters lacking a GCC box *cis*-element [18], recent studies demonstrated that ERFs can also bind different *cis*-elements such as VWRE (Vascular Wounding Responsive Element) [19].

ERFs are ubiquitous in plant kingdom and their functional implications have been studied in various plant species and in a wide range of processes including hormonal signal transduction [5], response to biotic [20,21] and abiotic stresses [22-24], regulation of metabolic pathways [25-28] and developmental processes [29-31]. Expression studies indicated that some ERFs are regulated by abiotic stresses such as wounding and salt stress [17,32,33] and more recently, it was demonstrated that ERFs are also involved in seed germination [3]. A number of studies demonstrated that in addition to regulating the expression of ethylene-responsive genes harbouring the GCC-box, ERFs can also regulate jasmonic acid and salicylic acid-responsive genes [20,34,35]. The presence of distinctive structural features among ERF classes suggests that different members of this family may display different functionalities and binding activities. The differential binding activities of ERFs might represent the mean by which ethylene signalling targets a specific set of genes thus providing the basis of the observed tissue and developmental specificity of plant responses to the hormone. However, structural characterization of ERF proteins has been restricted to *in silico* analysis and so far the structure/function relationship has been only superficially addressed [36].

The present study shows that the *ERF* gene family in the tomato is organised into 9 subclasses defined by distinct structural features. Based on functional analysis of 28 tomato ERFs and through testing their ability to activate or repress transcriptional activity of target genes, the data suggest that functional activity is conserved among ERF proteins sharing the same structural features. Moreover, data demonstrate that flanking regions of the core GCC-box sequence are part of the discrimination mechanism by which ERFs selectively bind to their target promoters. The data also show that *ERF* genes display tissue-specific patterns of transcript accumulation and uncover their regulation by auxin.

## Results

### Isolation and phylogenetic analysis of tomato ERFs

**Figure 1:**
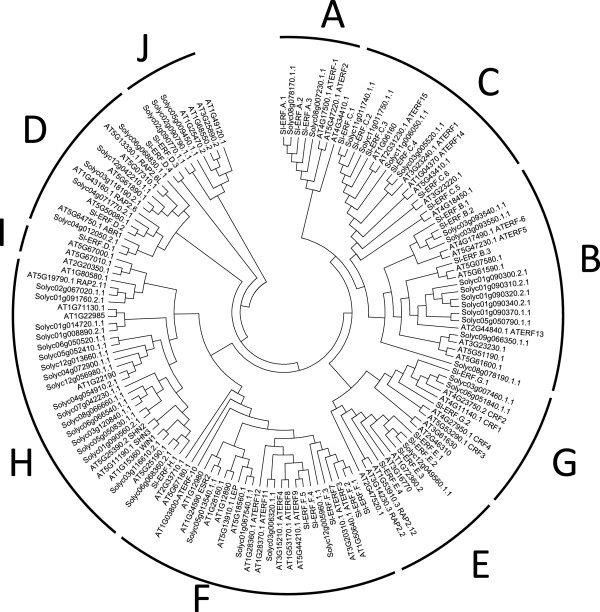
**Phylogenetic tree of Arabidopsis and Tomato ERFs.** Different subclasses are named by letters (**A** to **J**). Tomato genes for which the corresponding cDNA has been successfully isolated and that were subjected to functional analysis in this paper are named using the *Sl-ERF* nomenclature (Additional file 1) while other tomato *ERFs* are named using International Tomato Annotation Genome (ITAG 2.3) nomenclature. Phylogenetic trees were constructed with the whole protein sequences using neighbour joining method.

Tomato *ERF* cDNA clones were initially identified by TBLASTN search [37] in the tomato unigene database (http://solgenomics.net/) using a consensus sequence within the AP2/ERF domain as a query sequence. Forty nine unigenes were found from which AP2, RAV and DREB sequences were discarded based on their distinctive features regarding the number of AP2 domains, the presence of a B3-like domain and the presence of conserved amino acid residues, respectively. Using RACE-PCR extension approach, complete CDSs were obtained for 28 *ERF* unigenes that are representative of the main ERF sub-groups. Subsequently, building on the annotated whole tomato genome sequence recently released [38], a genome wide *in silico* screening allowed the identification of up to 146 genes encoding putative AP2-containing proteins distributed into 77 ERFs, 48 DREBs, 18 AP2 and 3 RAVs (Table 1). Since Arabidopsis ERFs have been classified so far using the AP2/ERF domain exclusively [10], we constructed the ERF phylogenetic trees using either the whole protein sequences or only the AP2/ERF domain. Due to the weak homology among ERF proteins outside the AP2/ERF domain, identical classification patterns were obtained with the two clustering methods (Figure 1). However, while 10 subclasses (A to J) define the ERF family in *Arabidopsis*, only nine are represented in tomato, which lacks subclass I (Figure 1, Table 2). The distribution of the tomato ERF proteins into nine individual subclasses is further supported by the presence within the AP2 domain of distinctive motifs and amino acid signatures specific for each subclass previously described in *Arabidopsis*[13] . In the absence of a consensual nomenclature and due to a lack of rational naming of ERF genes across plant species, we attempted to rename the tomato genes by giving a letter (A to J) to discriminate between different subclasses and a number to distinguish between members within the same subclass (Table 3). While complying with the most complete classification available in Arabidopsis [13] the proposed nomenclature better clarifies the correspondence between ERF subclasses in different species. The correspondence between the classification adopted here and those previously proposed for tomato ERFs [17,39] and for Arabidopsis ERFs [10,13] is given in Table 2. In this newly proposed classification class XI, defined by Sharma et al. 2010, splits into two subclasses I and J as proposed by Nakano (2006) [13] clearly demonstrating that Arabidopsis subclass I has no representatives in the tomato. Of particular note, members of subclass I harbour imperfect AP2 domain and comparatively to other subclasses is under-represented in Arabidopsis while it is missing in the tomato. Like in *Arabidopsis*, the overwhelming majority of tomato *ERF* genes (59 out of 77 genes) are intronless (Table 4). However, while Arabidopsis *ERFs* can bear at most a single intron, among the 18 tomato intronic genes, 5 have two introns. *ERF* intronic genes are found in four subclasses in Arabidopsis while they are spread across 6 subclasses in the tomato (Table 4). Although tomato *ERF* genes could be localized on 12 chromosomes, they present an uneven distribution with Chromosome 1 and 3 bearing 13 and 11 genes, respectively (Table 5). Fine mapping of *ERFs* on the tomato genome revealed that these genes could be distributed individually or in clusters. In particular, chromosome1 contains a cluster of 5 ERF genes (Solyc01g09300, Solyc01g09310, Solyc01g09320, Solyc01g09340, Solyc01g09370) that likely result from tandem duplication events (Table 5) as suggested by their position within sub-group B in the neighbourhood phylogenetic tree (Figure 1). The correspondence between the nomenclature proposed here for tomato Sl-ERFs and that issued by the ITAG 2.3 nomenclature (38) is provided in Additional file 1.

**Table 1:** Summary of the tomato AP2/ERF superfamily

**Classification**	**Number**
AP2 family	18
DREB family	48
ERF family	77
RAV family	3
Total	146

**Table 2:** The distribution of members of the ERF gene family among subclasses constructed in the present study and in previous classifications

	**Tomato**		**Arabidopsis**	
(This study)	[17]	[39]	[13]	[10]
A	I	IX	IXa	B3
B	III	IX	IXb	B3
C		IX	IXc	B3
D		X	X	B4
E	IV	VII	VII	B2
F	II	VIII	VIII	B1
G		VI	VI	B5
H		V	V	B6
I		XI	Xb-L	B6
J		XI	VI-L	B6

**Table 3:** Correspondence between the proposed nomenclature of tomato ERFs and previous nomenclatures

***New name***	***Previous name***	***Reference***
Sl-ERF.A.1	-	
Sl-ERF.A.2	LeERF1	[17]
Sl-ERF.A.3	Pti4	[40]
Sl-ERF.B.1	-	
Sl-ERF.B.2	-	
Sl-ERF.B.3	LeERF4	[17]
Sl-ERF.C.1	JERF2/TERF1	[41]
Sl-ERF.C.2	-	
Sl-ERF.C.3	-	
Sl-ERF.C.4	TSRF1	[42]
Sl-ERF.C.5	Pti5	[40]
Sl-ERF.C.6	ERF5	[43]
Sl-ERF.D.1	-	
Sl-ERF.D.2	-	
Sl-ERF.D.3	-	
Sl-ERF.D.4	-	
Sl-ERF.E.1	LeERF2	[17]
Sl-ERF.E.2	JERF1	[42]
Sl-ERF.E.3	JERF3	[44]
Sl-ERF.E.4	-	
Sl-ERF.F.1	-	
Sl-ERF.F.2	-	
Sl-ERF.F.3	-	
Sl-ERF.F.4	-	
Sl-ERF.F.5	LeERF3	[17]
Sl-ERF.G.1	-	
Sl-ERF.G.2	Pti6	[40]
Sl-ERF.H.1	-	

Reference to the first paper describing the gene is given in the last column.

**Table 4:** Presence of intron on tomato ERFs

**ERF gene**	**Class**	**Intron numbers**
Solyc01g008890	H	2
Solyc01g065980	E	1
Solyc03g116610	H	1
Solyc03g118190	D	1
Solyc03g123500	C	2
Solyc04g012050	D	1
Solyc04g051360	D	1
Solyc04g054910	H	2
Solyc04g071770	D	1
Solyc06g063070	A	2
Solyc06g065820	H	1
Solyc06g068360	H	1
Solyc06g068830	D	2
Solyc09g075420	E	1
Solyc12g042210	D	1
Solyc12g049560	E	1
Solyc12g056590	D	1
Solyc12g056980	H	1

**Table 5:** Distribution of ERFs on tomato chromosomes

**Chromosomes genes per cluster**	**Number of ERFs genes**	**Clusters of ERFs/ERFs**
Ch01	13	1/5
Ch02	5	2/2
Ch03	11	1/3
Ch04	6	
Ch05	9	1/3
Ch06	8	
Ch07	4	
Ch08	5	1/3
Ch09	4	1/2
Ch10	3	
Ch11	3	1/2
Ch12	6	
Total	77	8 clusters

### Subcellular localization of tomato ERFs

**Figure 2:**
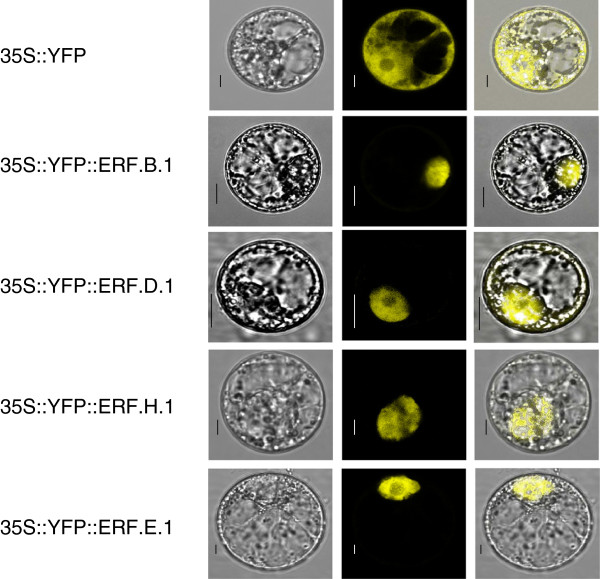
**Subcellular localization of Sl-ERFs.** ERF.B.1, ERF.D.1, ERF.E.1 and ERF.H.1 proteins were fused to the YFP (Yellow Fluorescent Protein) in the N-terminal region and the chimerical proteins were transiently expressed in BY-2 tobacco protoplasts under the control of the 35S promoter. Subcellular localization was then analyzed by confocal laser scanning microscopy. The merged pictures of the yellow fluorescence channel (middle panels) and the corresponding bright field (left panels) are shown (right panels). Control cells expressing YFP alone are shown in the top panel. The scale bar indicates 10 μm.

All ERF proteins display at least one canonical nuclear localization signal with the exception of ERF.B.1, D.1 and H.1 that lack any predictable nuclear targeting motif (Additional file 2). Since nuclear import of transcription factors is instrumental to their transcriptional activity, we investigated the subcellular localization of these three tomato ERFs by generating N-terminal fusions with the YFP expressed under the 35S promoter (35S:: YFP-ERF). Transient expression in tobacco BY-2 protoplasts coupled to confocal microscopy analysis clearly indicated that ERF B.1, D.1 and H.1 are exclusively targeted to the nuclear compartment similar to ERF.E.1 which contains a consensus nuclear localization signal (Figure 2).

### Relationship between structural features and functional activity of ERFs

**Figure 3:**
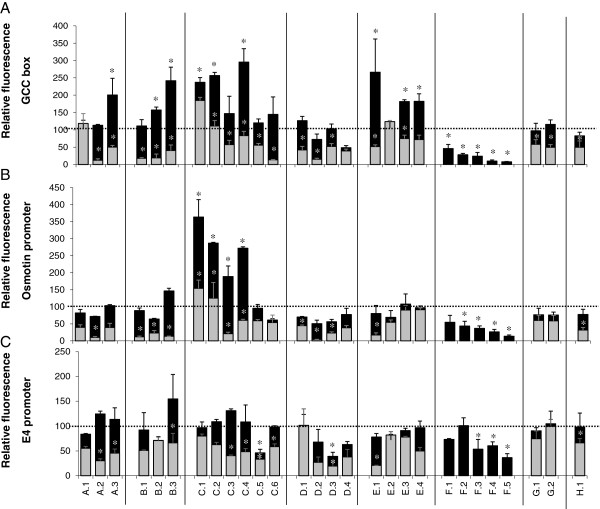
**ERF-mediated transcription from native and synthetic promoters.** Transient expression in single cell system has been used to assess the transcriptional activity of ERF proteins from different subclasses. The fluorescence of the reporter gene was measured by flow cytometry upon co-transfection with a reporter construct (GCC:: GFP or Sl-Osmotin Promoter:: GFP or Sl-E4promoter:: GFP) and an effector construct (35S::Sl-ERF or 35S::Sl-ERF-SRDX). The basal fluorescence obtained in the assay transfected with the reporter construct and an empty effector construct was taken as reference (100 % relative fluorescence). (**A**) ERF activity on synthetic promoter containing 4 direct repeats of the GCC-box. (**B**) ERF activity on osmotin native promoter containing the canonical GCC motif. (**C**) ERF activity on E4 native promoter lacking the GCC motif. The results are mean of 3 independent biological replicates. Analysis of variance (ANOVA) was performed to determine the effect of the subclass on ERF activity (p<0.05). The Mann–Whitney test indicates significant result (p<0.05). Black and gray Bars indicate relative fluorescence obtained with native or repression version of each ERF, respectively. Bars indicate SE of the mean.

To address whether specific structural features affect the ability of ERFs from different subclasses to drive the transcriptional activity of target promoters, a transient expression assay in a single cell system was used. Tobacco BY-2 protoplasts were co-transformed with effectors constructs carrying the ERF coding sequences driven by the 35S constitutive promoter and reporter constructs consisting of the GFP coding sequence driven either by a GCC-rich synthetic promoter or a native ethylene-responsive promoter. To discriminate between the situation where the absence of activity is due to the lack of binding to the target promoter from those where the ERF binds but remains neutral on the promoter activity, we used chimerical constructs as effectors consisting of ERF coding sequences fused to the SRDX repressor motif [45]. Since the dominant repression activity of the chimerical construct is mediated by epigenetics mechanisms [46], the absence of repression with any of the ERF-SRDX constructs can be interpreted as resulting from the incapacity of the ERF to recognise the target promoter. Figure 3A shows that ERF proteins can not only act as activators or repressors, but can also be neutral on the ethylene-responsive promoters. Experiments carried out with the SRDX fusion demonstrate that all ERFs have the ability to bind the GCC box containing promoter except ERF.A.1 and ERF.E.2. Members of subclass C display the strongest activation activity whereas ERFs from subclass F show the highest repression activity (Figure 3A). ERFs from subclass A, B and E are weak activators on the GCC box whereas those from class G and H are neutral (Figure 3A).

To test whether the regulation of the ethylene-responsive promoters by ERF proteins is strictly dependent on the presence of a canonical GCC motif, tomato osmotin and E4 native promoters containing or lacking a conserved GCC motif, respectively, were fused to the GFP coding sequence and used as reporter constructs. The data obtained with the fused SRDX repressor motif suggest that up to 16 ERFs out of the 28 tested in this study (Figure 3B) are capable of binding the osmotin promoter (p<0.05). In line with the results obtained with the synthetic GCC promoter, the osmotin promoter is strongly activated by ERFs from subclass C and repressed by ERFs from subclass F. By contrast, the transcriptional regulation of the native osmotin promoter by several ERFs from other subclasses did not match their behaviour with the synthetic promoters. Notably, the native osmotin promoter contains a number of *cis*-elements that are likely to drive the transcriptional activity in a more complex way than that displayed by the synthetic promoter containing simply the GCC-box. Overall, ERFs showing the strongest effect on the synthetic GCC promoter also display the most significant effect on the osmotin complex promoter. E4 promoter is a well described ethylene-inducible promoter lacking the conserved GCC motif that represent a potential target for ERFs. The data presented in Figure 3C indicate that ERF.C.5, ERF.D.3, ERF.F.3, ERF.F.4 and ERF.F.5, are capable to bind the E4 promoter and to repress its activity, suggesting that some ERFs can be active on ethylene-responsive promoters lacking the canonical GCC box *cis*-element.

The data reveal that ERF proteins are more active on the synthetic GCC promoter than on native complex promoters. The activity of the ERFs on the GCC box (Figure 3A) indicate that members of the same sub-class tend to modulate the activity of the target promoters in the same way (ANOVA p<10^-5^). Half of the ERFs tested (14 out of 28) have significant effect on the synthetic promoter while only 8 ERFs are active on the native osmotin GCC-containing promoter and 4 on the native E4 lacking a conserved GCC motif.

### The impact of the GCC box flanking regions on the binding affinity of ERFs

**Figure 4:**
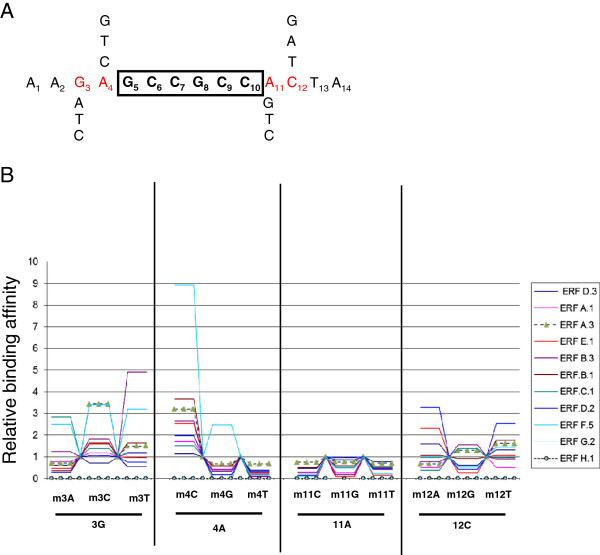
**Binding affinity of ERFs to the GCC-box is impacted by the nucleotide composition of the flanking regions.** (**A)** To assess the role of the nucleotide composition of the close environment of the GCC box, nucleotides flanking the chitinase GCC box were mutated. Different mutated GCC boxes were used as probe in gel shift assay to test the binding affinity of ERFs. (**B**) Binding affinity of ERFs to the mutated probes. Relative affinity is calculated with non mutated Sl-Chitinase (Solyc10g055810.1) as reference. The data are mean of 3 independent replicates. Analysis of variance with the R package reveals that the flanking region of the GCC box is significantly involved in the affinity of the binding (p<0.05).

We have previously demonstrated [17] that ERF proteins can display differential affinity to various promoters containing the highly conserved GCC-box. Hence, the hypothesis that the nucleotide environment of the GCC box may impact the binding affinity of the ERFs was tested. A total of 11 ERFs representing all subfamily types were challenged with the native Sl-Chitinase GCC box (5^′^-A_1_A_2_G_3_A_4_GCCGCCA_11_C_12_T_13_A_14_- 3^′^) or with mutated versions of this motif in gel shift assay experiments (Figure 4A). The four nucleotides flanking the GCC motif (G_3,_ A_4_, A_11_ and C_12_) were mutated and the binding of the ERFs was tested by EMSA and assessed by phosphoimaging. Mutation of A at position 4 (A_4_) into T or G dramatically decreased the affinity to the GCC box of all ERFs except that of ERF.F.5 (Figure 4B) while the substitution of A_4_ by a C increases the affinity of all tested ERFs. Strikingly, mutation of A_11_ to any of the three other nucleotides resulted in a dramatic loss of affinity of ERFs to the GCC box. These data suggest that nucleotides upstream (position 4) and downstream (position 11) to the GCC box impact the binding affinity in the same way for all the ERFs tested. By contrast, substitution of G_3_ or C_12_ by another nucleotide resulted in different effect on the binding affinity depending on the ERF tested (figure 4B).

### Expression pattern of *ERF*s

**Figure 5:**
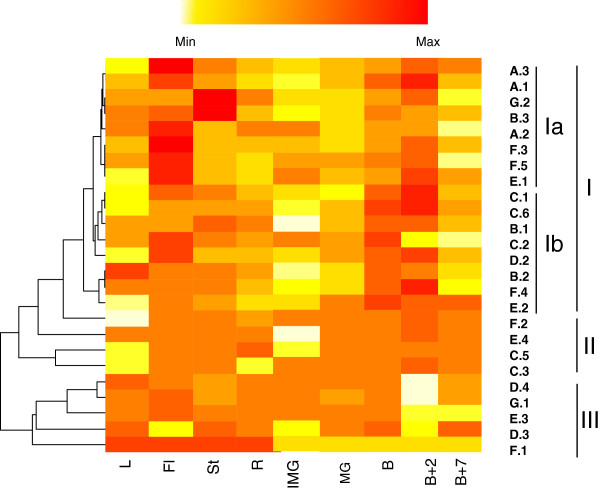
**Heatmap representation of the expression of ERF genes in different tomato tissues.** The data obtained by quantitative RT-PCR correspond to the levels of ERF transcripts in total RNA samples extracted from Roots (R), Leaves (L), Stem (St), Flower (Fl), Early Immature Green (IMG), Mature Green (MG), Breaker (B), Breaker + 2 days (B+2), Breaker + 7 days (B+7). The data presented correspond to 3 independent biological repetitions. Red and white colours correspond to high and weak expression of the ERF genes, respectively. Heat map was generated using R software.

To assist with the elucidation of the physiological function of tomato ERFs, we sought to gain more insight on the spatio-temporal expression at the transcriptional level of each member of the gene family. Transcript accumulation was assessed by qRT-PCR for 25 ERFs whereas for the remaining three, transcripts could not be detected in the nine different plant tissues tested. The heatmap representation of the global expression pattern allowed the clustering of tomato ERFs in three main clades (Figure 5), with clade I (16 ERFs) corresponding to genes preferentially expressed in reproductive tissues, clade II (4 ERFs) corresponding to genes with an ubiquitous expression without tissue preference, and clade III (5 ERFs) mainly active in vegetative tissues. Subclade Ia displays a distinctive expression pattern associated with the breaker +2 and flower stages, while subclade Ib contains genes whose expression is more pronounced at the breaker + 2 and breaker stages. These results suggest that some ERFs operate specifically in vegetative tissues (clade III) or reproductive organs (clade I), whereas few ERFs seem to be ubiquitous and might be involved in the development of various organs (clade II). Interestingly some ERFs are strongly expressed in B+2 and Flowers stage (clade Ia) suggesting a role in fruit set and during ripening while others seem to be active more specifically at the onset of ripening (Breaker and Breaker + 2, clade Ib). These data also point out to the absence of a clear relationship between the structurally defined ERF subclasses and their tissue type expression.

### Ethylene and auxin regulation of *ERF* genes

**Figure 6:**
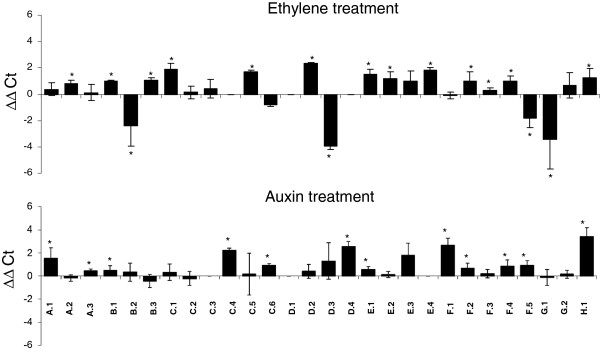
**Expression of ERFs in response to ethylene and auxin treatments.** Quantitative RT-PCR of *ERF* transcripts in total RNA samples extracted from 5-days dark growing seedlings treated for 5 hours with ethylene or with IAA for 3 hours. ΔΔCt refers to the fold of difference in *ERF* expression relative to the untreated seedlings. Stars indicate a statistical significance (p<0.05) using Mann-Withney test.

It has been reported that beside their regulation by ethylene, *ERF* genes can also be induced by other hormones among which auxin [47]. To test the responsiveness of tomato *ERF* genes to ethylene and auxin, transcripts accumulation in seedlings treated with ethylene or auxin for 5 and 3 hours, respectively, was assessed by qRT-PCR. *E4* and *SAUR*, known as ethylene and auxin-responsive genes, respectively, were used as controls to validate the hormone treatment. Transcript accumulation levels (Figure 6) indicate that 13 tomato *ERFs* are significantly induced (p<0.05) by ethylene and 4 significantly (p<0.05) down-regulated by this hormone. Interestingly, the expression of up to 12 *ERFs* is also positively regulated by auxin. Among these, 4 *ERF* genes (*ERF.B.1, Sl-ERF.E.1, Sl-ERF.F.4,* and *Sl-ERF.H.1*) are up-regulated by both hormones whereas *ERF.F.5* undergo opposite regulation by auxin and ethylene. More surprisingly, 6 *ERF* genes (*ERF.A.1, ERF.A.3*, *ERF.C.4, ERF.C.6, ERF.D.4* and *ERF.F.1*) are significantly up-regulated by auxin while they are not responsive to ethylene.

## Discussion

A wide range of developmental processes are known to rely at least partly on ethylene action, yet, the molecular mechanisms underlying the diversity of plant responses to this hormone are still awaiting some in depth characterization. The present study brings new insight on structural features that impact the binding affinity of Ethylene Response Factors (ERFs) to ethylene responsive promoters. Upstream of ERFs, the ethylene signal propagates via a linear transduction pathway which can hardly account for the wide range of ethylene responses. In its downstream part, ethylene signalling leads to the activation of ERFs that are encoded by one of the largest multigene family of transcription factors [7], and these transcriptional mediators may therefore represent one of the main step where the diversity and selectivity of ethylene responses can take place. The structural and functional characterisation of tomato ERFs carried out here provides some clues to the means by which ethylene selects target genes that are required to bring into play the appropriate physiological responses in a tissue-specific and developmentally defined manner. Taking advantage of the recent release of the complete annotated tomato genome sequence [38], a genome wide *in silico* screening identified 146 genes encoding putative AP2-containing proteins distributed into 77 ERFs, 48 DREBs, 18 AP2 and 3 RAVs (Table 1). Subsequently, the functional analysis concentrated on 28 members of the tomato ERF family encompassing 8 of the 9 subclasses of the gene family.

The data indicate that all ERF proteins display a clear nuclear localization, even though three members of the family lack any predictable nuclear targeting motif suggesting that their import to the nucleus may occur by a non-conventional localization signal or they might be conveyed to this compartment via an interaction with a yet unknown nuclear localized partner.

The large diversity among ERFs is well illustrated by the presence of distinctive motifs that are sufficient to discriminate between different subclasses. Recent studies already suggested a link between the structural classification of ERFs and their physiological function [13], yet, this hypothesis has not been supported by strong experimental data at the level of whole ERF family. It was reported that within class E, the conserved specific N-terminal domain, initially described in tomato ERFs [17], is dedicated to an oxygen dependent sequence of post-translational modifications that leads to degradation of the protein under aerobic conditions. In hypoxia condition, the RAP2.12 ERF protein is relocated from plasma membrane to nucleus to activate gene expression associated with hypoxia acclimation [48]. These results suggest that members of the subclass E are involved in hypoxia response.

Transient expression assays indicated that the activity of ERFs on synthetic GCC box promoter correlates well with their structural classification. That is, ERFs from subclass C are strong activators and those belonging to subclasses A, B and E are weak activators, whereas members of subclass F are clear repressors. By contrast, ERFs from subclasses G and H display no activity when challenged with the GCC box. Noteworthy, ERFs show weaker activity on the native complex osmotin promoter than on the synthetic promoter. This may be due to the high basal expression displayed by the native osmotin promoter in the absence of ERF effectors, which likely minimizes the induction effect observed in the presence of ERFs (Additional file 3). Moreover, the experimental data show that ERFs can regulate transcription from both types of ethylene–responsive promoters either containing (osmotin promoter) or lacking (E4 promoter) the GCC-box thus revealing their capacity to bind *cis*-elements without the canonical GCC motif. While it cannot be ruled out that ERFs may induce indirectly the *E4* promoter through the activation of primary target genes encoding transcriptional proteins active on the *E4* promoter, our data suggest that ERFs can interact with other *cis*-acting elements beside the canonical GCC motif. This is in agreement with recent studies demonstrating that, in addition to the GCC box, ERFs can bind *cis*-acting elements such as VWRE and GCC-like [19,49-51]. The clustering of the ERF proteins according to their activity on the GCC box, indicates that members of the same subclass tend to modulate the activity of the target promoters in the same way (Figure 3A). While the data show that the repressor and activator activities of ERFs correlate well with their structural classification, the recognition of the GCC-box by a particular ERF seems to depend on the promoter used and might be impacted by the nucleotide environment surrounding the *cis*-acting element.

Indeed, gel shift assay experiments show that the environment of the GCC box greatly impacts the affinity of ERF proteins to the conserved *cis*-element. In particular, the data (Figure 4) clearly demonstrate that the nature of the nucleotides at position N_3_ and N_12_ can have different and sometime opposite effect on different ERFs, thus suggesting that these bases contribute to the binding specificity of various ERFs to target promoters. Taking together these results suggest that the two nucleotides directly flanking the GCC box at position N_4_ and N_11_ are essential for the binding of any ERF to the GCC-box and, therefore, their nature determines whether or not the *cis*-element is functional for the binding to an ERF. By contrast, the nature of nucleotides at position N_3_ and N_12_ can either enhance or reduce the binding affinity, which supports their contribution in determining the binding specificity of various ERFs to target promoters. Previous work carried out with native promoters already stressed the putative role of the GCC box flanking regions in impacting the binding activity of the ERFs [17]. The same study also postulated that variation in amino acid composition within the binding domain may also impact the binding affinity of ERFs to their target promoters [17]. It was also postulated that the conserved motifs lying outside the DNA binding domain may also contribute to the differential affinity displayed by ERF proteins towards the GCC box-containing promoters. Recent study demonstrated that the *trans*-activating activity of some ERFs was localized in the acidic domain [52]. In keeping with this observation, ERF.D.1 and ERF.D.2 are lacking the acidic activator domain and accordingly are unable to activate the synthetic GCC box promoter. On the other hand, subclasses A, B, C and E ERFs harbour one or more acidic domains, however, not all of them display transcriptional activation of the synthetic promoter. Members of class F have an EAR repressor domain in the C-terminal region of the protein and accordingly they are all repressors of the activity of the GCC box-containing promoters.

Unravelling the expression pattern of *ERF* genes provides important clues towards the elucidation of their physiological function since physiological effects of transcriptional mediators not only depend on their activation or repression function, but also on their specific pattern of expression. While consistent with previous description concerning the expression of some ERF genes like *Pti4*[53] and *ERF1-4*[17], the data distinguish different ERF groups depending on whether they are preferentially expressed in vegetative, or reproductive tissues or whether they show ubiquitous expression with no tissue preference. Noteworthy, structural subclasses did not display tissue or organ specialisation as exemplified by the fruit development process throughout which ERFs from structurally distinct subclasses are expressed from flower anthesis to fruit ripening. This could be in line with the hypothesis that different responses to ethylene taking place in different tissues and at different developmental stages are mediated by different ERF proteins. Tissue and abiotic stress expression pattern of tomato ERFs has been already reported [39], however, the hormone-dependent expression remained unknown. As expected, a high number of *ERF* genes are ethylene-responsive with 13 *ERFs* being up-regulated and 4 down-regulated upon ethylene treatment. Less expected, our study revealed that up to 12 tomato *ERF* genes were regulated by auxin among which 6 undergo double regulation by ethylene and auxin while the remaining 6 others are exclusively auxin-responsive. It was already reported that, beside ethylene, *ERFs* can be regulated by salicylic acid and jasmonic acid [20,35,54], but the auxin regulation of such a high number of ERF genes has not been reported. The auxin regulation of the ERFs shown to be active on ethylene responsive promoters, suggests their potential contribution to the cross-talk between the two hormones. The phytohormones auxin and ethylene are essential regulators of plant development and it is well documented that ethylene and auxin regulate common physiological aspects such as hook formation [55,56], root hair differentiation [57], root elongation [58], root growth [59], and hypocotyl phototropism [60]. The mechanisms underlying the interactions between the two hormones are becoming better understood even though only few molecular players of this cross-talk have been identified so far. Besides acting independently on the same target genes, ethylene and auxin can mutually regulate each other’s biosynthesis and response pathways. In support to this idea, we previously reported that the down-regulation of Sl-*IAA3*, encoding a tomato transcriptional regulator from the Aux/IAA type, results in phenotypic responses related to classical auxin and ethylene-regulated processes [61,62]. Therefore, uncovering the auxin responsiveness of some members of the *ERF* gene family, may define new actors potentially involved in the cross-talk between the two hormones. Global transcriptomic analyses of tomato lines under-expressing *Sl-IAA9,* another tomato Aux/IAA gene [63,64], revealed the altered expression of a high number of ethylene-related genes, further supporting the idea of an active link between auxin and ethylene signalling during the flower to fruit transition. In keeping with the complex role of ERFs in mediating various hormone responses, ERF.A.1 and ERF.D.4 that are highly expressed in mature flowers, display strong up-regulation by auxin but not by ethylene. On the other hand, ERF.A.2 and ERF.C.1 are up-regulated by ethylene but not by auxin, and they are strongly expressed in the flower. Interestingly, many ERFs are expressed in flower and at early ripening stages (B and B+2), suggesting their putative auxin-dependent role in fruit set and fruit ripening process.

## Conclusion

The present study provides some molecular clues on how ERFs can contribute to the specificity and selectivity of ethylene responses through (i) the differential expression of the gene family members, (ii) the ability to negatively or positively impact transcriptional activity and, (iii) the capacity to select with specificity their target genes based on the nucleotide environment of the GCC-box. Considering the diversity of their transcriptional activity and expression patterns, *ERFs* possess the necessary features for channelling ethylene signalling to a selected set of genes required for the appropriate developmental responses or the desired responses to environmental cues. The insight gained in this study opens new prospects towards assigning a specific role for individual ERFs in controlling developmental processes and for identifying the direct target genes for each member of the ERF family.

## Methods

### Plants growing

Tomato (*Solanum lycopersicum cv* MicroTom) plants were grown in climatic chamber. The conditions are the following: 14-h-day/10-h-night cycle, 25/20°C day/night temperature, 80 % hygrometry, 250 μmol.m^-2^.s^-1^ irradiance.

### Cloning of over expressing and repressing ERF constructs

The full length CDS were obtained by RACE using BD SMART^TM^ RACE cDNA Amplification kit (Clontech, http://www.clontech.com) and the complete ERF CDS were, cloned in over-expression vector, with or without SRDX fusion in C-terminal [45] (Additional files 4, 5).

### Ethylene and auxin treatments

Five day-old dark-grown seedlings were treated with air or ethylene gas (50 μL/L) for 5 hours and RNA was extracted from the corresponding tissues. Auxin treatments were performed on 7 day-old light-grown seedlings by soaking (3 hours) in auxin-containing (20 μM) or auxin-free MS solution. Three independent biological repeats were performed for each experiment.

### Analysis of ERF gene expression

RNA samples were obtained from different plant tissues: Immature Green fruit (17 days post anthesis), Mature Green fruit (1 day before Breaker), Breaker fruit, Breaker + 2 days, Breaker + 7 days, leaf, flowers, roots and stem. Real-time quantitative PCR was performed according to Pirrello et al. 2006 (Additional file 6). Heat map representation was performed using centring and normalized ΔCt value, with R software to visualize clustering.

### Phylogenetic analysis

In order to identify *Sl-ERF* genes in the tomato genome, the AP2/ERF domain of a tomato Ethylene Response Factor sequence (GenBank number NP_001234308) was used as BLAST query sequence against the tomato ITAG2.30(Sl2.40) protein database . The same database was also screened by an HMM analysis using a typical AP2/ERF domain PF00847 as query. One hundred forty six genes were identified as possibly encoding proteins containing the AP2/ERF domain (Table 1). The evolutionary history was inferred using the Neighbour-Joining method [65]. The optimal tree with the sum of branch length = 13.53632771 is shown. The phylogenetic tree was linearized assuming equal evolutionary rates in all lineages [66]. The tree is drawn to scale, with branch lengths in the same units as those of the evolutionary distances used to infer the phylogenetic tree. The evolutionary distances were computed using the Poisson correction method [67] and are in the units of the number of amino acid substitutions per site. All positions containing gaps and missing data were eliminated from the dataset (Complete deletion option). There were a total of 63 positions in the final dataset. Phylogenetic analyses were conducted in MEGA4 [68]. Conserved motifs were determined using MEME version 3.5.5 [69].

### Transcriptional activity tests by transient expression in a single cell system

A synthetic reporter construct (4XGCC-GFP) was generated by fusing a synthetic promoter containing 4 GCC-box repeats upstream of the minimal −42 to +8 TATA box from the 35S promoter of *Cauliflower mosaic virus* to the coding region of the Green Fluorescent Protein (GFP). Reporter constructs were also generated with native promoters, *E4* (S44898) and *Sl-Osmotine* (C08HBa0235H18.1), fused to GFP. Effectors constructs were generated by fusing the 35S promoter to the CDS of the ERF genes. For transient assays, tobacco (*Nicotiana tabacum*) BY-2 protoplasts were co-transformed with reporter and effector constructs [61]. Transformation assays were performed in three independent replicates. After 16 h, GFP expression was analysed and quantified by flow cytometry (FACS Calibur II instrument, BD Biosciences) on a flow cytometry platform (IRF31). Data were analysed using Cell Quest software. For each sample, 100–1000 protoplasts were gated on forward light scatter and the GFP fluorescence per population of cells corresponds to the average fluorescence intensity of the cell population after subtraction of autofluorescence determined with non-transformed BY-2 protoplasts. The data were normalized using an experiment with protoplasts transformed with the reporter vector in combination with the vector used as the effector plasmid but lacking the Sl-ERF coding sequence.

### Subcellular localization

YFP N-terminal fusions were obtained with ERF.B1, D.1, H.1 and E.1 and used for tobacco protoplast BY-2 transfection according to Chaabouni et al. 2009 [61]. The subcellular location of the fluorescence was determined after 20 hours using confocal microscopy.

### Electro mobility shift assay

Proteins used for the gel shift assay were produced in vitro using a “TNT®T7 Quick kit for PCR DNA” (Promega, http://www.promega.com). Primers used for amplification of ERFs were design according to manufacture recommendation and include Kozak T7 sequences. Gel retardation experiments were performed with the GCC-box probe mutated in the flanking nucleotides according to the procedure described in Tournier et al. 2003 [17] (Additional file 7). Scanning and radioactivity quantification were achieved by a “Phosphoimaging Fujifilm Bas 5000” and the software “Image Gauge” (Fuji Film, http://www.fujifilm.com).

### Sequences

Coding DNA sequences of the 28 studied ERFs are provided in Additional file 8.

## Authors’ contribution

JP and BCNP participated in the design of the study, performed the experiments, and contributed to the writing of the manuscript. WZ contributed to the cloning of *ERF* genes and to qRT-PCR experiments. KC participated in the design of the project. IM carried out the transcriptional activity experiments in the single cell system. MZ contributed to the *in silico* analysis of the *ERF* gene families. AL and JCP participated in the ethylene and auxin treatment experiments. MOT contributed to the design of the SRDX-repressor motif experiments. FR contributed to the design of the study and of the experiments, to bioinformatics analysis and to drafting the manuscript. MB conceived the study, and participated in its design and coordination and contributed to the writing of the final manuscript. All authors read and approved the final manuscript.

## Supplementary Material

Additional file 1 **Correspondance between *Sl-ERF *nomenclature and ITAG 2.3 nomenclature.** Click here for file

Additional file 2 **Schematic diagram of protein structure of the isolated 28 tomato ERFs defining the 8 subclass.** Each colored box represents the AP2/ERF domain and conserved motifs, as indicated below the diagram. The position of the motif is indicating by the number on the top of the diagram. The name of motif by Nakano is given inside the box [13] Click here for file

Additional file 3 Comparative basal activity of the synthetic promoter (4 X GCC) and the native promoters, fused to the GFP in the absence of added ERF effectors. Click here for file

Additional file 4 Primers used for RACE amplification. Click here for file

Additional file 5 **Primers used for CDS amplification.** Each pair of primers encompasses the complete CDS from the ATG to the STOP codon. Adaptors attB1 and attB2, have been added at the 5′ end of sense and antisense primers, respectively. Click here for file

Additional file 6 **Primers used for qRT-PCR.** Primers designed with PRIMER EXPRESS software in sequence specific regions for each ERF. Specificity was further checked using BLAST against all tomato unigenes (Tomato unigene database). Click here for file

Additional file 7 Primers used for the synthesis of radioactive GCC box probes utilized in EMSA experiments. Click here for file

Additional file 8 Coding sequences of the 28 studied ERF. Click here for file
