# Supplementary figures and images for: Functional analysis and binding affinity of tomato ethylene response factors provide insight on the molecular bases of plant differential responses to ethylene
Source: BMC Plant Biol. 2012 Oct 11;12:190. doi: 10.1186/1471-2229-12-190 (PMC3548740; doi:10.1186/1471-2229-12-190)

**Class A**

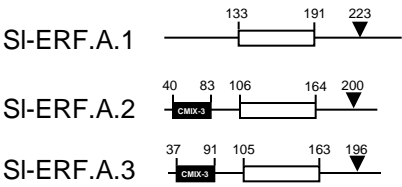

**Class B**

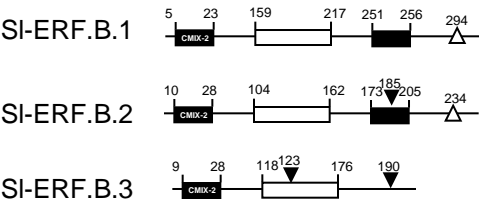

**Class C**

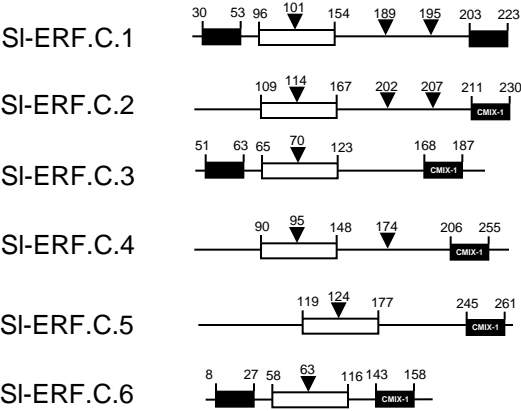

**Class D**

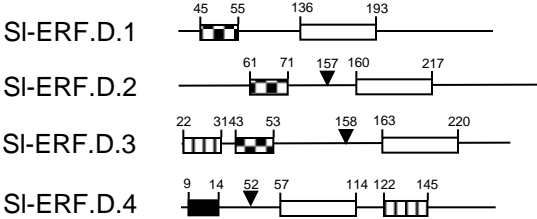

**Class E**

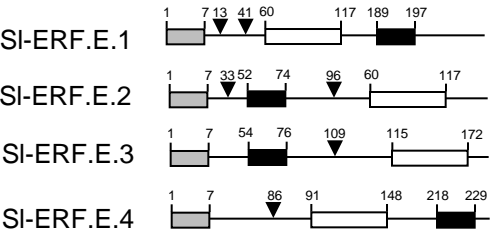

**Class F**

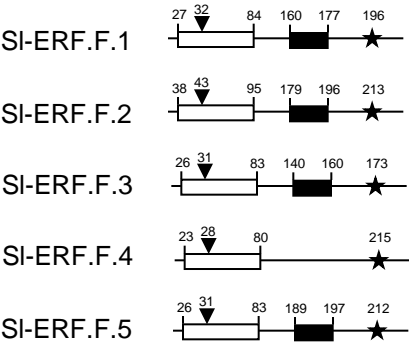

**Class G**

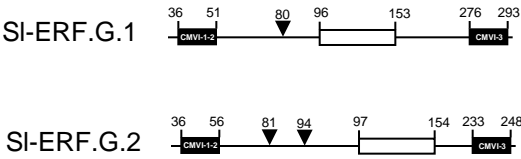

**Class H**

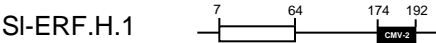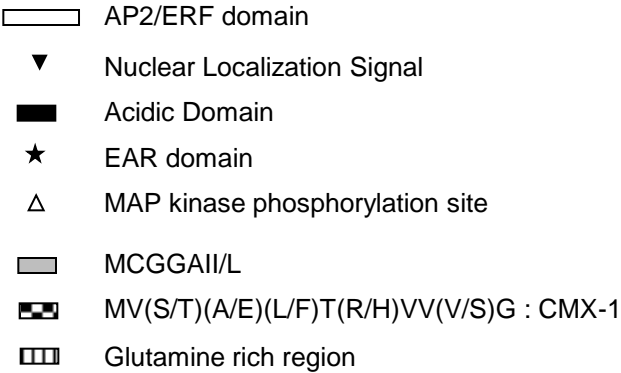

Supplement: Additional file 2 — Schematic diagram of protein structure of the isolated 28 tomato ERFs defining the 8 subclass. Each colored box represents the AP2/ERF domain and conserved motifs, as indicated below the diagram. The position of the motif is indicating by the number on the top of the diagram. The name of motif by Nakano is given inside the box [13] [file 1471-2229-12-190-S2.pdf]

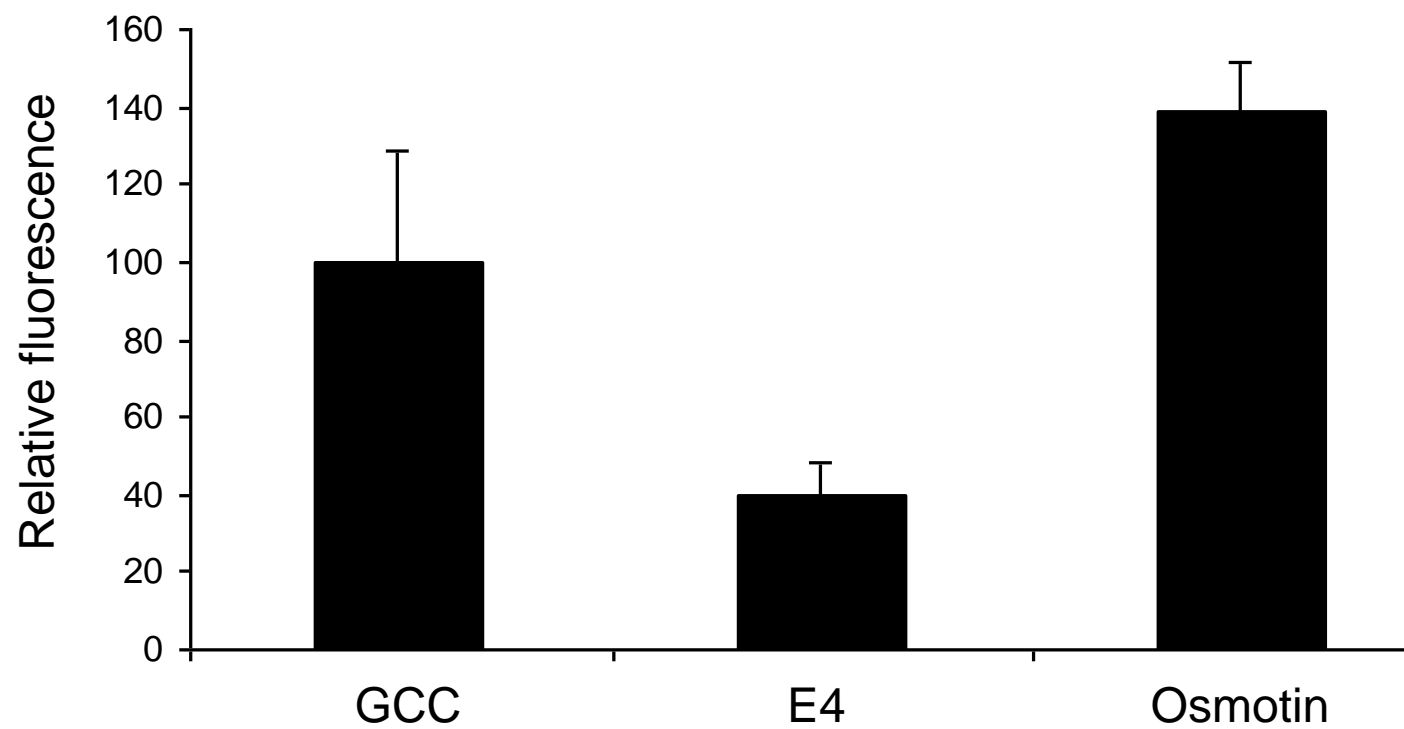

Supplement: Additional file 3 — Comparative basal activity of the synthetic promoter (4 X GCC) and the native promoters, fused to the GFP in the absence of added ERF effectors. [file 1471-2229-12-190-S3.pdf]
