# Supplementary material for: Functional analysis and binding affinity of tomato ethylene response factors provide insight on the molecular bases of plant differential responses to ethylene
Source: BMC Plant Biol. 2012 Oct 11;12:190. doi: 10.1186/1471-2229-12-190 (PMC3548740; doi:10.1186/1471-2229-12-190)
Supplement: Additional file 8 — Coding sequences of the 28 studied ERF. [file 1471-2229-12-190-S8.docx]

>Sl-ERF.A.1

atgtattcaaattgtgaactagaaaatgatttttcagtactcgaatcaattagaagatacttacttgaagattgggaagctccattaacgagctctgaaaactcaacatcctcagagttcagccggagcaacagcattgaatccaatatgtttagtaattcatttgattatacacctgaaatttttcaaaatgatattcttaatgaaggatttggatttggatttgaattcgagacttctgattttataatccctaaattagagtcacaaatgtcaatcgaatcacctgaaatgtggaatttaccggaatttgtggctccattagagacggcggcggaggtgaaagttgaaacaccggttgagatgacaactacgacgacgaagccaaaggcaaagcattatagaggtgtgagagtgaggccatgggggaaattcgcggcggaaattagagatccggcgaaaaatggagcacgagtttggctcggtacatatgagacggcggaggatgcggcgttggcttacgacaaggcggcttttcgcatgcggggatcacgtgcattgctgaattttccgttgaggattaattccggtgaaccggatcctgttagagttggatcgaagagatcgtcaatgtcgccggagcattgttcatcggcgtcgtcgacgaagaggaggaagaaggttgctcgtggaacaaagcaataa

>Sl-ERF.A.2

atgtatcaacttcccacttctactgagttaactttttttccggcagaattcccggtgtattgccggagttcaagtttcagtagtctcatgccatgtttaaccgaatcatggggtgacttgccgttaaaagttaacgattccgaagatatggtaatttatgggtttctacaagacgcttttagtatcggatggacgccgtcaaatttaacgtccgaggaagtgaaactcgagccgagggaggagattgagccagctatgagtacttctgtttctccgccgacagtggctccagcggctttgcagcctaaaggaaggcattacaggggcgttagacaaaggccatggggaaaatttgcagcggaaataagagatccggctaaaaacggcgcacgggtttggcttggaacttacgagtcggctgaggaagccgcactcgcttatgataaagccgcttttaggatgcgcggtactaaggctctattgaatttcccgcatagaattggtttaaatgagccggagccggttagagtgacggttaagagacgattatctgaatcggctagttcatcggtatcatcagcttcggaaagtggctcgcctaagaggaggagaaagggtgtagcggctaagcaagccgaattagaagttgagagccggggaccaaatgttatgaaagttggttgccaaatggaacaatttccagttggcgagcagctattggttagttaaaatatggagctaa

>Sl-ERF.A.3

atggatcaacagttaccaccgacgaacttcccggtagattttccggtgtatcgccggaattcaagcttcagtcgtctaattccctgtttaactgaaaaatggggagatttaccactaaaagtcgacgattccgaagatatggtaatttacggtctattaaaagacgctctaagcgtcggatggtcgccgtttaatttcaccgccggcgaagtaaaatcggagccgagagaagaaattgaatcgtcgcctgaattttcaccttctccggcggagaccacggcagctccggcggctgaaacaccgaaaggaagacattatagaggcgttagacagcgtccgtgggggaaatttgcggcggagattagagatccggcgaagaacggagctagggtttggcttggaacgtacgaaacagctgaagaagctgcaattgcttatgataaagctgcttatagaatgagaggatcaaaagcacatttgaatttcccgcaccggatcggtttgaatgaaccggaaccggttcgagttacggcgaaaaggcgagcatcgccggaaccggcaagctcgtcgggaaacggttccatgaaacggagaagaaaagccgttcagaaatgtgatggagaaatggcgagtagatcaagtgtcatgcaagttggatgtcaaattgaacaattgacaggtgtccatcaactattggtcatttaa

>Sl-ERF.B.1

atggattcttcttcactagaaatgataagacaacatcttcttgatgatgttgttttcatggaaacttgttcttcttcttcttcttcttcattagaaacaacaagtagtacactttattctcaaacctcatcgaattcggaatctttagaatcattaacctctgagatcaaacttgaaagcaatttctctgtttatcctgatttcatcaatacacctcaaagttcaaatcttgaatctgtctctcgttttttcgataactcaactattgaattccaagctaaaccccaaaagaaaagaagtttcaatgatcgaaaaccttcgttaaacatttcgattccttctgttaagaaaacagaggaaccaaaaacaggggaagtaaaaacaggggaaccaaaaacagaggagccaaaaacaggggaagtgaaaacagagtactctgttaaggagaaaatggtagaaaattcggagaaaaagcgatacagaggagtgagacaaaggccatgggggaaatttgcagcggagattcgtgacccaactagaaaggggacacgagtttggttaggaacattcgatactgcaatggatgcagccatggcatatgacagagcagcatttaggctcagagggagtaaagcaatcttgaattttccacttgaggtaagcaatttcaagcaagaaaatcatgagattgagaaaaatgttgtgaatttgaattcgaatacgaattcttgtgggaaaagggtgagaggggaaatggagaatgatgacggaattgtaatgaagaaagaggtgaaaagagaacaaatggtggcaactccattaacaccttcaaattggtcttcaatttgggattgtggaaatggaaaaggtatttttgaagtgccacctttgtcaccattatcaccacattcaaattttggttattctcaacttttggtatcatag

>Sl-ERF.B.2

atgggttctccacaagagacttgtacttcacttgatttgattaggcaacatctttttgatgaatctctggaccagacttgtttctcttttgaaacaactcaaacttcaaatcttgatgacatcgcaagcttctttaatgctacttcaaaaacagagtatgatggttttttcgaatttgaggcaaaacgacatgttatccgttcaaattctccgaaacagagtaacttgagagaacggaagccatctctgaacgtagcaataccggcgaagcctgttgttgttgtagagaacgttgagattgagaagaagcattacaggggagttagacagaggccatgggggaagtttgcagcggagattcgtgacccaaatagaaaggggactcgagtttggttaggaacatttgatactgctgtggatgcggcaaaggcatatgacagggcagcgtttaagcttagaggaagcaaagcaatattgaatttcccactcgaagttgcaaactttaagcaacaaaatgatgagactaaaacagagatgaagtcgtcaggcagtaaaagggtgagaggagaaacagaggaattagtaatcaagaaggaaaggaaaatagaagaagaaagagtactcccaacggcggcggctccattaacaccgtcaagttggtcgacgatttgggatgaaaagggtatttttgaggtgccaccattgtcaccattatctcagcttgttatgatataa

>Sl-ERF.B.3

atgacgaaacaagatgaaggattaacattagaactcatacgacaacatctcctcgaagatttcacaactacagaatcattcatcgacagtctcaattcttgtttttccgatcacatctcctcctccgatgacatctcccctgttttcacttcagtaaaaacagagccatctacatccaattccctctcagattcacccaattcctcatacccaaatgaacccaactccccaatttcccgttacttcaatctccgctccgatttccctgaattcaaaatcgattcagataccatcctcagtccagttttcgacagctccgccggttctaatgaagacaataataagaagaagaattacagaggggtaaggagaaggccatgggggaaatttgcggcggagataagagatccaagtcgaaaaggatcgaggatttggttgggtacttttgatactgatattgatgctgctagagcttatgattgtgcagcgtttaagatgagaggaagaaaagctattctgaattttccgttggatgccggaaaatctggtgctccggcgaatgttggccggaaaaggagaagagagaacaagatggagttggtgtag

>Sl-ERF.C.1

atgtcaagcccactagagatagatacttcattttcacattccaatttgttgtttttggaagatgaatcatcatggagtaatactcatgatccatttgttgatattgatgaatatctaccaataattataccatgtaatgatgaagaaatagtagtagaatcctcaaacactagtactacaacaacaacaacaacaacatcaaaagtagcaagtatccaaaatattcatcatgatcaagaagaggtaacatccatagagaaaaaacatgaagatgatcaagaaaaacattatattggagttagaaagaggccatggggtaaatatgcatcagaaattagggattcaacgcgtaatggaattagggtttggttaggaacatttgatactgctgaagaagctgctttagcttatgatcaagccgcattatcaatgaggggtccttggtctctactcaattttccaatggaacatgttaaaaaatctcttgaaaatattgagtattcttgtaaagatggattatctccagctgctgttttaaaagctactcataaaactagaagagtcaagcacaagagaagtagtagaaagaagaagaatgagaatttggaaaatgtttttgtttttcaagacttgggagttgaattattagaagagcttttaatgacttcatcatag

>Sl-ERF.C.2

Atggaatcttcatcccctaaaactcaatatccaaatttcaatttcttccaagatcaatcatcattaccatggaatgatcaacatttcttagatgaatatttgactaacatcgaccaaaacaacgatcattctctaccaggaagtacttgttcattcttaacctcgaaagaaagttatagacgggaagtttcctcctccaacctacatcaattaccaagaagttggtcatcctcaaacgatacgaattcctctaaagaaagcaataatcgtcatgaaatcgaagaggtcacgtctcatcaccatgataagaataactccaccaaacactacataggagttagaaagagaccatggggaaaatatgcagcggaaataagggattcaacaagaaatgggattagggtttggttaggaacatttaatacttgtgaagaagctgctttagcgtatgatcaagctgcacttacaatgagaggtccattggcacttttaaattttccaatggacaaagtaagagaatcacttgaaaatattaagtatatttgtgaagatgggatttcaccagctgctgttttaaaggcaacaaataaaatgagacgtgttaaacataagagaaataggaagaagagaaatgttttggtttttgaagatttgggtgctgaattgttagaggaacttttgacgagtacttcctctaattag

>Sl-ERF.C.3

Atggattattcatctcgggatgatcttctttttcactataattcacttccatttaacgttaacgatacacaagacatgttactttataatcttgttgctgaaggatcatcgcaagaaacagtgaattcgtcgtctagctatggaataaaggaagaggaagtgacctcatatgaagaagaaagaaaagataagaattacagaggtgttagaaagaggccatggggtaaatatgctgctgaaattcgtgattctacgaggaatggtgttcgtgtatggctaggtacatttgataatgctgaagaagctgcgttagcgtatgatcaagctgcatttgctatgagaggttcgatggccatacttaattttcctgtggagatagttaaggaatcgctaaatgaaatgaagtgtagatttgatggtaattgttcgccggtgattgaattgaagaagagatattcaatgaggagaaagagtgttagtagaaaaaacagagcaagaaaagatgttgtggtttttgaagatttgggtgctgagtatttagaggaattgttgatttcttcagaaagtatcacaaattggtga

>Sl-ERF.C.4

atggattcttcttcttcttcatctcaattcttctactcaatgaattctgatttaaattcatcagattcttcatacgaatggtccaatttcaacacacaatcttatctccctttcaacgtgaacgactccgaagagatgcttctcttcggagttcttaacgctgctcatgaagaaacaacatccgaaacagtcacatcgcatcgcgttaaagaagaagaagttacctcagaatccgaggttattgaagcaataccggcgaaggagaagtcgtaccgaggtgttaggaggcgtccatggggtaaattcgcagcggagataagagattctacgagaaatggggttcgagtatggttagggacatttgatagcgcggaagatgctgctttagcttatgatcaagctgcgttttcaatgaggggtaattctgccattttgaattttccagtggagaccgttagggattcgctacgtgacatgaaatgtcacgtagacgatgattgctcccctgtagtggcgcttaaaaagcgccactccatgaggaaaaggagcacgaattccaaaaaagttaatagtattagtaaagtagtgagggaagttaaaatggaaaatgtaaataatgtagttgtgtttgaagatttgggtgctgattatttagaacaacttttaagtagtagttcaagtgatcaaagtagttgtgatgcaacttattttagtccatggtaa

>Sl-ERF.C.5

atgagacattctttaaagatgactactcatcatgtggaaaataataatcaagaacaagatcaagtagcatgtgaagagattctagagaatgtttgggcaaacttcatatccaagaatgatcaaaattctcaaaaggtgacaaatgaatattgttgtgaacaatattgggaacaacttccaattcttgaaagattaccaagcttaggaagatggatatcaatgggagctgaaacttgggaagatattctcaatggaattattattccttctcataacaacgaaaattcaaacgacgaatcaacatgcaaagatgttgtcaacgttgagaagaaggaggagaagaagaagatggtgcattatagaggggtaagaaggaggccatggggaaaatacgcggctgagataagggattcatcaagaaaaggagctagggtttggctagggacatttagtactgctgaagaagctgctatggcttatgacaaggcggctttgagaattagaggtcccaaggcctatcttaattttccacatgaaatggttgctcaagctataggaatatctaatggtccttgtgaaaaagaatggaccttttcatcatcaagtcaatataattcaaggaaaagggtatcaagggattggaacatgtatgaaaatttggatgaaattaatcaattgcctatggagaagaaaattatgagaagcatggaggaggatctttttaatgacttggatatcctagagtttgaggatcttggaagtgattatttggatagtttattatcctctttgtaa

>Sl-ERF.C.6

atggttccaactcctcaaagtgatttacctcttaatgagaatgactcacaagagatggtattatatgaagttcttaatgaagctaatgctctaaatattccttatttaccccaacgaaatcaattactccctagaaataatattcttcgtccattacagtgcataggcaagaaatacagaggagtacgacgtcgtccgtgggggaaatacgctgcggaaattcgcgattcggctagacatggtgcgagagtatggctaggtacgttcgaaactgctgaagaagctgcgttagcttatgatagagcggcttttagaatgcgaggtgctaaggcactacttaattttccatctgaaatagtgaacgcctctgtttcagtagacaaattaagtttgtgctcaaatagttacactacgaataataattcagattcaagtttaaatgaagtttcaagtggaactaatgatgtatttgaatcaagatgttaa

>Sl-ERF.D.1

atgtgtaatattgttcactacaaggtggcgaattcgaatgataacaggagtagtagacaagacgatgaagggattaatgtgtttaatacgatgtttcaagggaatattaatagagaagaagaaatgtctgttatggtttctgcattaactcgtgttgttgttggtaatcatcctagtgaaaatatcgaaaatcatcatcaaaataatacattgatttctaggggtgttggagaaaaaagaggacgtgatgaagtattattacatggaactaattcttctcatatgatattatcatcaggtggtgaaggttcaagcattaggacaacaagagaagcaacattcatatacactaattcaacaaacaatagcattattgatgaatctgttaataatcaagtaagacgacgatacagaggagttagacagaggccatgggggaaatgggcagctgaaataagagatccatataaagcagcgcgtgtttggttaggtacttttgatactgctgaaggggctgctagagcttatgatgaagctgctcttacatttagaggtagtaaagcaaaactaaatttcccagaaaatgttacattattagtgccttcttcaattcaacaacccatttactcctcacccgacccagccatttctccgtatcgttccaattttattattggacatacctctacggaagtggagcctatacttcacaccaatccttcaaattttattgagcctatagctcacacttcatctctgtatcgttccaattttattgaacggaatcatcacatggtgcagcaggagccgtattttcaggcaggtagtactagtggtggaagtgattttcatcaaactacaaattcttctaattcgtcaatttatgatcacccttcttcttcatctggataa

>Sl-ERF.D.2

atgtgctttttaaaggtggcgaattcaagaaaatcaagtgaatttgttagatttacagatacagatgatacacaaaccaccgccgtgactgcgatcggcggcggtgttgaaggcggtggtcagtttgattattcgatgtattcagggtattgtgattctcaggcgagagatatgtcggagatggtgacggagtttacacgtgtggtatcgggtcaggattatcgacccgataccagatgttattcggttaattcaccgtctccggcttattcttcgtccagctcgggttctagagctggactgaagagaagccgtgatcaacaagaatttggaactgggttgtcatcttcttcctctgttaaaattgaagaagctacaagtatggttgcaccaattcccgctttcacaaccacaatcacaaccacgaccacaacaggtgagggttcgagcgaagaaacaggaggagataggaggaggaaatacagaggtgtacgacaacgaccatggggtaaatgggcagcggaaataagagatccacataaagccgccagagtttggttaggaacattcgatacagcagaagctgcagcaagagcatatgatgaagctgcattgagatttcgaggaaacagagcaaaactcaacttccctgagaacgccagattgtcatcgttaccacaaacacaaaatactgtaacgtcaacaatctccaatccatcccctctaatagctcaaccaacgtcgttcctcaatcctatccagagttcagatacaacaagagactactgggaatactcacaattgttgcaaaatccaggagattttacggatcaacaaccatcaaacttattggaacaaatgttcgttgcctcatcgatggcaatgttgcattcaaacacattgccattaatatcttcgtcttcatcgttagctacatcagcaacttcttcaacgtcatatcccctgttattttcgagttattacacaccacaaactaatcaaattcaaggaaccaacacaagtagcaccagcaccactagcagctcaagtttttctacaacattttggagtagctctagccaatatcctccatcttctagttaa

>Sl-ERF.D.3

atgcattggttaaataaaagatttagacaagaagcaggaatgaattcgaattcgaattccctccaaaataacaatcaatttcaacagcagcaaccaaggcttactggagatgaagagtactctgttatggttgcaactctgaaaaatgtgatcaatggtaatattccaacgcaaaattatcaagaattcaatgtcttttcgccatataattattctactgccaccacaaccacgaatgttacttcttcttcttcgccttctactagtatgtctactagtttcgaacaagtattgggtgtctctgctgaacaagaaccttgtcaattttgcagaattcaaggttgtttaggctgtgacatttttggtaccacattttcttcttcttcttcagcgcctgctgctgtggctgctcctgttgctgataataagaagaagagtagtagtagtagtacagctacagtcgcaatcgcgaagaaaaagaagaagaattacagaggagtgagacagaggccatgggggaaatgggcagcagaaattcgtgatcctcgaaaagctgcacgtgtttggctgggaactttcactacggcagaagaagcagctagagcttatgataaagccgccattgaattcaggggtccacgagctaaattgaatttttcatttgcggattataccgttgacactcaagaacaacagagcactttatcttcttcaccacaacaattaccagaagagcctcagcaatcccagacagcgaataataattccgattatggaaatgaaatttgggatcaattgatgggtgacaatgaaattcaagattggttgaccatgatgaatttcaatggcgactcttctgattctggcgggaatgttcacagcttttaa

>Sl-ERF.D.4

atgtcgccgcccttgtttcgcgtaccggaagaaacagagcgttgtcagtactgcaaaataaatggttgtttaggctgcaactattttgcaacctcatcagctgcagctggtgttgttaacaacaacaaggcattaaagattgttgggaagacgaaaaagaagaagaagaattacaggggagtgagacagagaccatggggaaaatgggcagcggaaattagagatccaagaagggcagctagagtatggcttggaacatttactacagctgaggacgcagctagagcttatgacagagcagctattgaatttagaggtccaagagctaagcttaatttctcatttacagattacacttcaattcaacaacacaatactactacacccatgcaagtgctgcaacaacaacaaccagctccctcgcagttacaacaaggaataaacacagaagaagaagagttctgggatcaattgatgaattcggacaatgaaattcaacattatctttatagagaatcatctgattctgctaatggctatattgctcatagcttctag

>Sl-ERF.E.1

atgtgtggtggtgcaattcttgctgatatcattcctcctcgtgaccgccgtttgtcatccaccgacctatggccgactgatttctggccaatttccacccaaaatgttcctctcaaccccaaacgagctcgaccctctacaggtggtgagcagatgaagaagaggcaaaggaagaatctttacagagggataagacaacgtccatggggtaaatgggctgctgaaattcgtgacccgagaaaaggggttagggtttggttaggtactttcaacactgctgaagaagctgcaagagcttatgatagagaagctcgtaaaatcaggggtaagaaagctaaagttaatttccccaatgaagatgacgaccattactgctacagtcatccagagccccctcccttgaacattgcttgtgatactactgttacttacaatcaagaatcaaataactgttaccccttttactcaatcgagaacgttgaacctgttatggaatttgcaagttataatggaattgaagatggaggagaggagatggtgaaaaatttgaataacagggttgtagaggaagaggagaaaacagaggatgaagtgcagatactttctgatgagctgatggcttatgagtcattgatgaagttctatgaaataccgtatgttgacgggcaatcagtggcggcgacggtgaatccagcggcggagaccgccgtgggcggtggctcgatggagctttggagttttgatgatgttagtcgtctacaaccaagttataatgtagtttaa

>Sl-ERF.E.2

atgtgtggtggtgcaattatctccgatttggtacctcctagccggatttctcgccggttaaccgctgattttctatggggtacatccgatctgaacaagaagaagaagaaccctagtaattaccactcaaagcccttgaggtctaagtttattgaccttgaagatgaatttgaagctgactttcagcacttcaaggataattctgatgatgatgatgatgtgaaggcatttggccccaaatccgtgagatctggtgattcaaactgcgaagctgacagatcctccaagagaaagaggaagaatcagtaccgggggatcagacagcgtccttggggtaagtgggcagctgaaatacgtgatccaaggaaaggtattcgagtctggcttggtactttcaattcagccgaagaggcagccagagcttatgatgctgaggcgcgaaggatcagaggcaagaaagctaaggtgaactttcctgatgaagctccagtgtctgtttcaagacgtgctattaagcaaaatccccaaaaggcacttcgtgaggaaaccctgaacacagttcagcccaacatgacttatattagtaacttggatggtggatctgatgattcgttcagttttttcgaagagaaaccagcaaccaagcagtacggcttcgagaatgtgtcttttactgctgtagatatgggactgggctcagtttccccttcagctggtacaaatgtttacttcagctctgatgaagcaagtaacacttttgactgctctgatttcggttgggctgaaccgtgtgcaaggactccagagatctcatctgttctgtcggaagttctggaaaccaatgagactcattttgatgatgattccagaccagagaaaaaactgaagtcctgttccagcacttcattgacagttgacggtaacactgtgaacacgctatctgaagagctatcggcttttgaatcccagatgaagttcttgcagatcccatatctcgagggaaattgggatgcatcggttgatgccttcctcaatacaagtgcaattcaggatggtggaaacgccatggacctttggtccttcgatgatgtaccttctttaatgggaggtgcctactaa

>Sl-ERF.E.3

atgtgtggtggttctataatctccgattacatagaccctagccggacttctcgccggctcaccgccgagtttctatggggtcgtttcgatctcggtaagaagcaaaaaaatcccaacaattatcactctaaagctaagcatttgcgatctgaagttgttgacgactttgaagccgattttcaggacttcaaagagttatccgatgatgaggatgttcaagtcgatgtcaagccatttgccttctctgcttccaaacactctactggttccaaatctttgaaaactgttgattcagacaaggatgctgctgctgataaatcctctaagagaaagaggaagaatcaatatagagggatcagacagagaccttggggtaagtgggcagctgaaatacgtgacccaaggaaaggggttcgggtctggctgggaaccttcaatactgcagaagaagctgccaaagcttatgatattgaggcgaggaggatcagaggcaagaaggctaaggtaaactttcctgatgaagctcccgcccctgcatcaagacacactgttaaggtgaatcctcagaaggtccttcctgaggagagcctgtattcacttcagtccgactcagcaatcatgaacagcgtggaggatgaccattatgattcttttggattttttgaagagaaacccatgacaaaacagtatggatatgagaatgggagcagtgcttctgcagatacgggatttggttcgttcgtcccttcagctggcggtgatatctacttcaactctgatgtaggaagcaactcttttgaatgctctgattttggttggggagagccatgctccaggactccagagatatcatctgttctgtcagctgctattgaatgtaatgaagctcaatttgttgaagatgccaattctcagaaaaagttgaaatcatgcaccaacaaccccgtagctgatgatggaaacccccgttactatggtacctga

>Sl-ERF.E.4

atgtgtggaggtgccataatctccgattatgatcccgccggaagcttctaccggaaactttctgctcgtgacctctgggctgagctggaccctatctccgactactggtcctcttcttcctcatcctcaaccgtcggaaaacctgattccgctctgtcgccggtgactcactccgtcgataagccaaataaatcagattccggcaaaaaaggtaataagactgtgaaggttgagaaggagaagagtagtggaccaaggccaaggaagaacaagtacagaggaataagacagaggccatggggaaaatgggctgctgagattcgcgatccacagaagggtgtacgcgtttggcttggtacattcaacacagcagaagatgctgctagagcctatgatgaggctgctaagcgcattcgtggtgataaggctaaactcaactttccagccccatcaccaccagctaagcgacagtgcactagcactgtcgctgctgctgatacaccaccagcactactccttgagagttctgacaactctcctttgatgaactttggatatgatgtccagtatcagagccaaactccctactaccccatggaaatgcccatagttagtgaagattatgaactgaaggaacagatttccaatttggaatcgttcctggaattggagccatctgatcaattttcagggatcgtcgattctgatcctcttaatgtttttctgatggaggactttgcttcaactcatcatcagttctactga

>Sl-ERF.F.1

atgagaagaggcagagcaactccggcggcggcggcggcggcggtgaagccagatggatctggaggattgaaggagattaggtttcgtggagttcggaagaggccatgggggagatttgctgcagagattagagatccatggaagaaaactagggtttggttaggtacttttgattcagctgaagatgctgccaaagcttatgatgctgcagctcgaactcttcgtggacctaaagctaaaactaatttccctttacctatgtattctcagcatcatcagttcaatcgaagtttaaaccctaatgatcggttagttgacccgagattgtactcacaagaagctccgatcatttgtcaaagacctacatcgagcagtatgagtagtactgtggaatcattcagtggaccgagaccgccacgtcagcaaacggcggttttgccttcgagaaaacatcctagatcgccgccggttgagccggatgactgcaggagtgattgtgactcatcgtcttctgttgttgaagatggtgattgtgaagggggaaatgacaacatcgtttcttcatctctcagaaatccactgcctttcgatctcaactttccacctccgatggatgatgtttatgctaattcaaatgatctttactgcacagcactatgtctttga

>sl-ERF.F.2

atgcggagaagcagagcagccgctgcggcgagacaagttccggcgacggaagttccggtaccggcaccggtggccggagaacacaacggatctggaggatctaaggagataaggttccgtggagttcgaaagagaccatggggaagatttgcagcagagattagagatccatggaagaaaactagggtttggttgggtacttttgattctgctgaagatgctgctcgtgcttatgatgcagcagctcgtactcttcgtggacctaaagctaaaactaatttccctttaccttcttctcatcatctacctccatatcctcaccatcatcagttcaaccaaagcatcaaccctaacgatccctttgtcgattcccggttatactctcaggaccacccattagtttcacagagacctacttcaagcagcatgagtagtacggtggagtccttcagtggaccacggcagccgccgcggcagcagacggcagcttccgtgccttccagaaagtatccccggtcaccgcctgttgtcccggacgattgccatagcgactgtgactcatcgtcttctgtcgttgaagacggtgaatgtgataacgacaacatcgcttcttcctctttcagaaagccgttgcctttcgatctaaacttaccggcaccgatggatgacttcagcgccgacgcatatgccgatgatcttcactgcacagcactatgtctttga

>Sl-ERF.F.3

atgcgccaccggaagtcgtcggagctgaaaagaccaggatctgacctccttcaacagcctgacgccgacccacctcgttatcgaggtgttcgtaaacggccatggggtagattcgcagcagagattagagatccgattaaaaagactcgagtttggctgggtacctttgacacagctgaagacgccgcacgcgcttacgacgatgctgcacgcgctctccgtggagctaaggcgaaaactaatttcaatatgttacctctaacagatgatccttatgatgatgagtttgagcttttccccaatccgagaccggcttctagcagtatgagcagtacgttggaatcgtctagtgggcctcgtggcggatcgagtagtaaggtgacccggatgaagattcctcgcccagttcgtccgatggaggaatgccggagtgattgcgattcgtcgtcgtctgtggtggatgatcggtgtgatgttgatcaaacgtcatcgtttgtgaccaaacaacctctgccgttcgatctgaatctgccacctccgtcggataacgatggagttgatgttgatgatttgcacgtcaccgctttatgcctctaa

>Sl-ERF.F.4

Atggctgtgaaagataaggctgtgaaaggaggtaatgtgaaagtgaatcatggagttaaggaagttcactacagaggtgtaaggaagaggccatggggtcgttacgctgcggagattcgtgacccgggtaagaagagtcgggtctggctgggtacttttgatacggcggaggaagcggctaaggcttacgatgccgctgccagagagtttcgtggacctaaagcgaagacgaatttcccctttccggcggagatgaataatgttggtaacaataacagtcagagcccgtgtgggagcagtaccgtggagtcatccagcggagaaacggttgttcacgcgcctaatacgcgacacgcgccgctggagctggatctcacgcgccgtctcggtgccgctgctgaaggtggacgtggaggtgtcggctacccgatcttacaccagcagccgacggtggcggttctgccgaacggtcagccggttttgttgtttgattctatgtggagaccgggagttgttagtaggccgtatcaggttgtaccggcgacgatggagtttgccggtgtcggtgccggagttgttactagtgtgtcggattcgtcttccgttgtggaagagaaacattatgggaaaaagggacttgatcttgatcttaaccttgcgccacctatggaagtttaa

>Sl-ERF.F.5

atggcgcctaaggaaaaaattggtgcagttacagctatggcaatggtgaatttaaatggaatttcgaaagaggtgcattatagaggtgtaaggaagaggccatgggggagatacgcggcggagattagagatcctgggaaaaaaagtagggtttggttaggtactttcgatactgcggaggaggcggctagagcttatgataacgctgctagagaatttcgtggagcgaaagcgaaaactaattttccgaaattagaaatggaaaaagaggaagatctgaaattcgctgtgaaaaatgaaatcaatcggagtccgagtcagactagtactgtggagtcatcgagtccggttatggttgattcatcatcgccgttagatctaagtctctgtggatcaatcggcgggtttaatcatcatacggttaagttcccgagctccggtggaggttttaccggttcggtacaggcggtgaatcatatgtactatatagaagcacttgcacgcgccggagttataaagttagaaacaaatcggaagaaaacggtagattacctcggtggtggtgactctgattcatcaacggtaattgattttatgcgtgttgacgtgaaatcaaccaccgccggtttaaatctggatctcaactttcctccaccggaaaacatgtga

>Sl.ERF.G.1

atggaatcacaaaaaatcaaaaagaaattagtccacaaaactatcactactaagtatgatcatcacaacaagtggactcctaaagttgttcggatttgttacactgattgtgatgctactgattcttcaagcgacgacgatgacgacgagaggaatcgagtgaaaaaatacgttacagagattaaatttgagaagaaaatggctgctgcagatgtgaggaaatcgttgaattcgaataagaagaagaagaaagcgatcgatttgaagagagatgagaatgttaaaaagtttcgcggtgtcagacagaggccatggggaaaatggtctgcggagattcgagatccggtgaaaaaaacgagggtttggttaggtacttttgataccgctgaagaagcggctatgaaatataatatagccgctattcaattgcgcggagctgatgctatcattaattttattgagacacctttcccaaaggaaaatgcgatcacttcagtatcggattatgattccacaggggaatgtgaaaacctctgttctcccacctcagttttgaggcagaataataacaataatgataaagataacgaagatgcgattgcgattgatactaaaattatgaacgatgagagcaaaaaaatggaaatggatgaaaatggatttatgtttgatgataatttgccattaatggatcagagtttccttaaggatttcttcgattttcgatccccttctccattgatggatgatgtattattaccaggttttagcgatggaatgggattattaccagaagtgttgagtattcatggaaatagaatgttggatgaagatttggagacttgtaagtgggcaaatgatttcttccaagatgtttgttga

>Sl-ERF.G.2

atgacggaaaattcagttccggtgattaaattcactcaacacatagtaactacaaacaagcatgttttttctgagcataacgaaaaatccaattcagagttacaaagagttgtgaggattatacttacagatgccgatgctacagattcttccgatgatgaaggccggaatactgtacggagagtgaagaggcacgtgacggagatcaaccttatgccgtcaaccaaatcgatcggcgacagaaaacgaagatcggtgtctccggattctgacgtcactcgtcggaaaaagtttagaggcgttcgtcaaagaccgtggggtcgttgggctgcagagattcgggacccgaccgggggaaaacgggtgtggttgggtacttatgacaccccagaagaagcagctgtcgtttacgataaagctgcagttaagctcaaaggtcctgacgccgttaccaattttccggtatcaacaacggcggaggtaacggtgacggttacggaaaccgaaaccgagtctgttgccgacggtggagataaaagcgaaaacgatgtcgctttgtcacccacctcagttctctgtgacaatgattttgcgccgtttgacaatctagggttctgcgaagtggatgcttttggtttcgacgttgattcacttttccggctgccggattttgctatgacggagaaatactacggcgatgaattcggcgaatttgactttgacgattttgcccttgaagctcgatag

>Sl-ERF.H.1

atggctagggcacaacaaagatatcgaggagttcgacagagacattggggttcttgggtctccgaaattcgccatccattgttgaagacaagaatttggttaggcacttttgagacagcagaagatgcagcaagagcatatgatgaagcagcaaggctaatgtgtggtccaagagctagaactaatttcccatacaacccaaacatgccacaaacatcttcctctaagctactctcaactacattaacagccaagttacacaaatgctacatggcttcacttcaaatgaccaaaacctcaccacaaggacaaaaattagcaaaaaatgcaaccaatgttcaagaaagtgttattaattcctataaaatgaaacaacaaatgttggtaccaaagccatcagtactattgactcatcatgatcatcatgaggaagctaaagtagtcaacttgggagtgggagtaattaggaaagttgaagatcaagtacttgagggtataccacaatttgtcaagccacttgaagatgatcacattgaacaaatgattgaagaattgttggattatggatccattgagctttgctctaatgttgttccttctcaccaaatccagtga
